# miR-134 in extracellular vesicles reduces triple-negative breast cancer aggression and increases drug sensitivity

**DOI:** 10.18632/oncotarget.5192

**Published:** 2015-09-24

**Authors:** Keith O'Brien, Michelle C. Lowry, Claire Corcoran, Vanesa G. Martinez, Melissa Daly, Sweta Rani, William M. Gallagher, Marek W. Radomski, Roderick A.F. MacLeod, Lorraine O'Driscoll

**Affiliations:** ^1^ School of Pharmacy and Pharmaceutical Sciences & Trinity Biomedical Sciences Institute, Trinity College, Dublin, Ireland; ^2^ Cancer Biology and Therapeutics Laboratory, Conway Institute, UCD School of Biomolecular and Biomedical Science, Dublin, Ireland; ^3^ Leibniz Institute DSMZ, German Collection of Human and Animal Cell Cultures, Braunschweig, Germany

**Keywords:** exosomes/extracellular vesicles, miRNAs, breast cancer

## Abstract

Exosomes (EVs) have relevance in cell-to-cell communication carrying pro-tumorigenic factors that participate in oncogenesis and drug resistance and are proposed to have potential as self-delivery systems. Advancing on our studies of EVs in triple-negative breast cancer, here we more comprehensively analysed isogenic cell line variants and their EV populations, tissues cell line variants and their EV populations, as well as breast tumour and normal tissues. Profiling 384 miRNAs showed EV miRNA content to be highly representative of their cells of origin. miRNAs most substantially down-regulated in aggressive cells and their EVs originated from 14q32. Analysis of miR-134, the most substantially down-regulated miRNA, supported its clinical relevance in breast tumours compared to matched normal breast tissue. Functional studies indicated that miR-134 controls STAT5B which, in turn, controls Hsp90. miR-134 delivered by direct transfection into Hs578Ts(i)_8_ cells (in which it was greatly down-regulated) reduced STAT5B, Hsp90, and Bcl-2 levels, reduced cellular proliferation, and enhanced cisplatin-induced apoptosis. Delivery *via* miR-134-enriched EVs also reduced STAT5B and Hsp90, reduced cellular migration and invasion, and enhanced sensitivity to anti-Hsp90 drugs. While the differing effects achieved by transfection or EV delivery are likely to be, at least partly, due to specific amounts of miR-134 delivered by these routes, these EV-based studies identified miRNA-134 as a potential biomarker and therapeutic for breast cancer.

## INTRODUCTION

Triple-negative breast cancer (TNBC) is responsible for 15–20% of breast cancers and it accounts for a disproportionate number of breast cancer deaths. Poor outcome corresponds with the innate aggressiveness of TNBC, augmented by the lack of targeted treatments [1–4]. Platinum-containing agents are among the drugs showing some benefit in TNBC [5, 6]. Anti-Hsp90 drugs also show promising results [7, 8], although some pre-clinical studies indicate TNBC to be less sensitive to Hsp90 inhibitors than HER2-overexpressing tumours [9–11]. Recently, microRNA (miRNA) profiling has been used in an attempt to discover TNBC sub-classifications [12], as well as to identify biomarkers or therapeutics for TNBC.

miRNAs [13] regulate a plethora of cellular processes including apoptosis, proliferation and differentiation [14], are commonly down-regulated in cancers and have relevance as biomarkers and therapeutic potential as tumour suppressing agents in many cancers [15, 16], including breast cancer [17–19]. Exosomes contain miRNAs [20] and it has been established that miRNAs can be transferred from cell-to-cell by exosomes, subsequently mediating epigenetic alterations in recipient cells [21]. It has also been shown that exosomes can be manipulated to transfer miRNAs representing therapeutics to recipient cells *e.g*. by acting in combination with VEGF inhibitors in leukaemia treatment [22].

Previously using a TNBC cell line (Hs578T) and its aggressive clonal variant (Hs578Ts(i)_8_) as models systems, we investigated the potential of exosomes/microvesicles (collectively termed extracellular vesicles/EVs) to influence the phenotype of “recipient”/secondary cells. We also assessed effects of EVs isolated from TNBC patients’ sera compared to those from healthy volunteers. These results indicated that the EVs released by the more aggressive cells carried the same traits to all secondary cell lines analysed (*i.e*. increasing their proliferation, migration, and inducing neovascularisation/angiogenesis), in a manner indicative of the innate phenotypes of the cells of origin. Additionally, EVs from TNBC patients’ sera, compared to those from healthy controls, increased the invasiveness of recipient breast cancer cells [23].

Here we chose to commence our studies with the same isogenic cell line variants (Hs578T and Hs578Ts(i)_8_) and their corresponding EVs. Hs578T and Hs578Ts(i)_8_ have the same genetic background, which make them an interesting comparison model. However, Hs578Ts(i)_8_, compared to Hs578T cells, have a more aggressive phenotype. Specifically they have 2.5-fold higher migratory capacity, 3-fold higher invasive (through extracellular matrix) capacity, and form 25 times more colonies in soft agar. Furthermore Hs578Ts(i)_8_, unlike Hs578T, produce tumours *in vivo* in nude mice [24]. This cell line pair is, therefore, very useful for investigating the comparative capabilities of EVs to transfer phenotypic traits representative of their cell of origin to secondary recipient cells. So, advancing on our previous studies, here we profiled the miRNA content of EVs to potentially identify mediators of the EV-induced signals and questioned whether the EVs could be manipulated into transporting miRNAs of choice to secondary cells, to both decrease cell aggression and to increase their sensitivity to anti-cancer drugs. From this, we have identified loss of miR-134 in cells and their EVs to be associated with increased cellular aggressiveness. Our functional studies support miR-134′s potential use as a therapeutic agent in TNBC, through its targeting of STAT5B [25] to subsequently reduce Hsp90 [26] and Bcl-2 expression, ultimately adding value to anti-cancer agents.

## RESULTS

### Isolation of EVs from Hs578T and Hs578Ts(i)_8_ conditioned media

**Figure 1:**
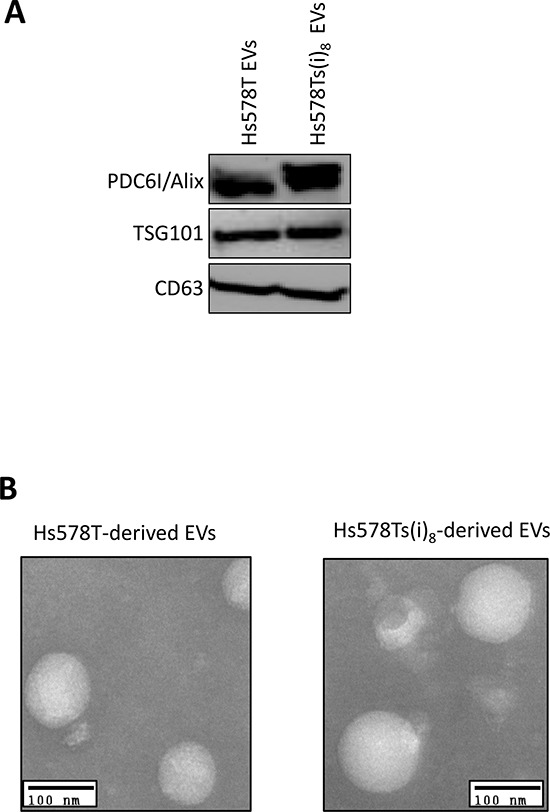
Confirmation of successful isolation of nano-sized extracellular vesicles (EVs) from Hs578T and Hs578Ts(i)_8_ conditioned medium **A.** Immunoblot analysis confirmed the presence of exosomal markers PDC6I/Alix, TSG101 and CD63 on analysis of the vesicles isolated from medium conditioned by the Hs578T and Hs578Ts(i)_8_ cells. **B.** Transmission electron microscopy showed these to typically be nano-sized vesicles of approximately 30–100 nm in diameter (scale bar: 100 nm).

Using procedures we previously reported [23] and that have also been extensively applied by Umezu *et al*. [22], EVs were isolated from medium conditioned by Hs578T and Hs578Ts(i)_8_ cells. To confirm that our isolates had hallmarks of EVs (as we previously described [23]), the presence of three exosomal markers *i.e.* PDC6I/Alix, TSG101 and CD63 were verified (Figure 1A). Transmission electron microscopy (TEM) confirmed that our isolates were of the expected 30–100 nm in diameter, indicative of exosomes. However, here we use the term extracellular vesicles/EVs as the presence of some microvesicles cannot be completely ruled out.

### miRNA profiling of Hs578T and Hs578Ts(i)_8_ cells and their respective EVs

**Figure 2:**
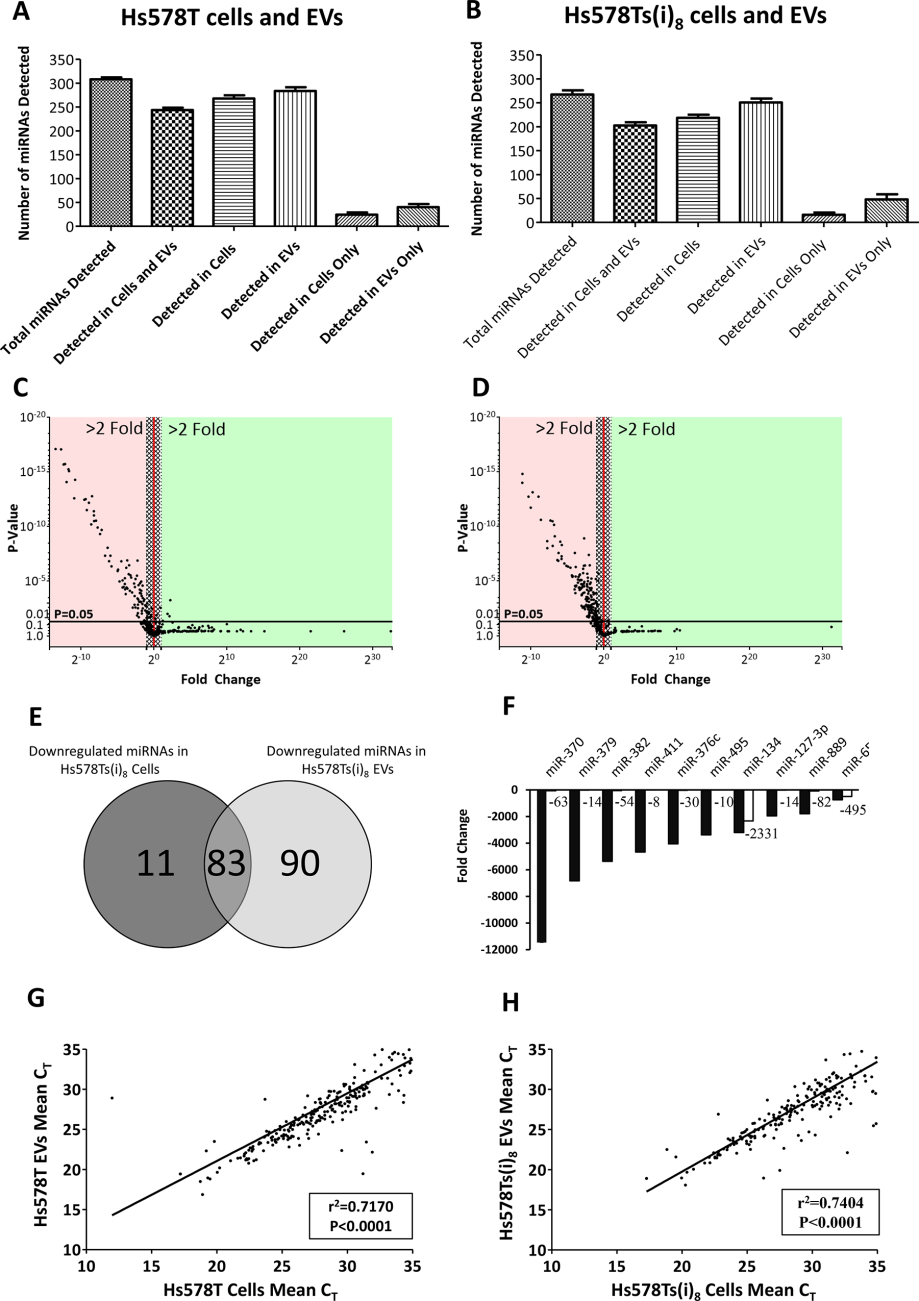
miRNA contents of Hs578T and Hs578Ts(i)_8_ cells and their respective EVs Following miRNA profiling using low density arrays representing 384 miRNAs, the numbers of miRNAs detected in **A.** Hs578T cells and Hs578T EVs, and **B.** Hs578Ts(i)_8_ cells and Hs578Ts(i)_8_ EVs were calculated and plotted. **C.** The spread of up- and down-regulated miRNAs in Hs578Ts(i)_8_ *versus* Hs578T cells, and in **D.** Hs578Ts(i)_8_ EVs compared to Hs578T EVs, noting that no miRNAs were at significantly increased levels in Hs578Ts(i)_8_ EVs. **E.** Numbers of miRNAs which were commonly down-regulated in both Hs578Ts(i)_8_ cells and their EVs *versus* Hs578T cells and their EVs, respectively. **F.** Fold changes for the ten most substantially down-regulated miRNAs in Hs578Ts(i)_8_ cells (*black*) and their corresponding EVs (*white*) when compared to Hs578T cells and their EVs. **G.** Linear regression analysis showing the correlation between the miRNA C_T_ values in Hs578T cells and EVs, and **H.** Hs578Ts(i)_8_ cells and EVs. Graphs represent triplicate biological repeats and are displayed as mean ± SEM.

To identify miRNAs that are substantially altered in the more “aggressive” Hs578Ts(i)_8_ cells and corresponding Hs578Ts(i)_8_ EVs, compared to the parent cell line (Hs578T) and its EVs, we performed miRNA expression profiling on biological triplicates of each of these 4 populations. Considering both parent Hs578T and Hs578T-derived EVs, a total of 308 miRNAs were detected. As indicated in Figure 2A, 244 (79%) of these miRNAs were detected in both the cells and their EVs; 24 (8%) were detected in the cells only and 40 (13%) were detected in the EVs only. Similarly, for the Hs578Ts(i)_8_ cells and their EVs, a total of 270 miRNAs were detected in both the cells and EVs, 202 (75%) of these were in both Hs578Ts(i)_8_ cells and EVs with 16 (6%) in the cells only and 51 (19%) miRNAs detected in the EVs only (Figure 2B).

Distinct differences between the miRNA profiles of Hs578Ts(i)_8_ cells and Hs578Ts(i)_8_ EVs compared to their parental (Hs578T) counterparts were found. As shown in Figure 2C & 2D, there is an overall tendency for miRNAs to be down-regulated in the more aggressive Hs578Ts(i)_8_ cells and their corresponding EVs, when compared directly with Hs578T cells and EVs. Eighty-three miRNAs were commonly down-regulated in both Hs578Ts(i)_8_ cells and EVs compared to Hs578T and EVs, respectively (Figure 2E; see Suppl. Table 1 for all 83). One miRNA, miR-146b-3p, was at higher levels in both Hs578Ts(i)_8_ cells and EVs, but this was not statistically significant (2.17 ± 0.826; *p* = 0.229). Figure 2F represents the fold changes for the ten most substantially down-regulated miRNAs in Hs578Ts(i)_8_ cells. Of these, miR-134 was most substantially down-regulated in Hs578Ts(i)_8_ EVs *versus* Hs578T EVs. Linear regression analysis showed strong correlation between miRNAs detected in Hs578T cells and their EVs (Figure 2G), with Hs578Ts(i)_8_ cells and EVs showing a similar finding (Figure 2H).

### Validation of miRNA changes by qPCR

Five of the down-regulated miRNAs were selected for further validation by qPCR. These five miRNAs were selected based on the following criteria: (i) greatest significant fold change of down-regulation in both Hs578Ts(i)_8_ cells and their EVs compared to Hs578T cells and their EVs, respectively; (ii) potential functional relevance from target prediction software (TargetScan), and (iii) literature mining. If two or more miRNAs seemed to be of equal interest based on (i) and (ii), literature mining would be included to identify which miRNA analyses could potentially add the most to our understanding of TNBC. While qPCR analysis of miR-655 in Hs578Ts(i)_8_ EV samples did not confirm the significantly lower levels apparent using low density arrays, miR-134, miR-370, miR-889 and miR-376c were all confirmed to be at significantly lower levels in Hs578Ts(i)_8_ cells and its EVs when compared to Hs578T cells and EVs (Table 1).

**Table 1:** Validation of miRNA expression by qPCR

miRNA	Cells Fold Change(Mean ± SEM)	*P*-value	EVs Fold Change (Mean ± SEM)	*P*-value
**miR-134**	1872.11 ± 0.002	8.9 × 10^−11^	73.68 ± 0.005	3.0 × 10^−9^
**miR-370**	1244.39 ± 0.005	4.6 × 10^−9^	33.26 ± 0.02	4.6 × 10^−7^
**miR-889**	565.66 ± 0.001	2.5 × 10^−22^	25.69 ± 0.02	9.8 × 10^−7^
**miR-376c**	2546.46 ± 0.002	5.7 × 10^−11^	14.33 ± 0.02	1.2 × 10^−6^
**miR-655**	537.42 ± 0.001	6.5 × 10^−12^	0.98 ± 1.58	4.5 × 10^−1^

Results represent three biological repeats

### The ten most down-regulated miRNAs in Hs578Ts(i)_8_ cells and EVs originate from 14q32

**Figure 3:**
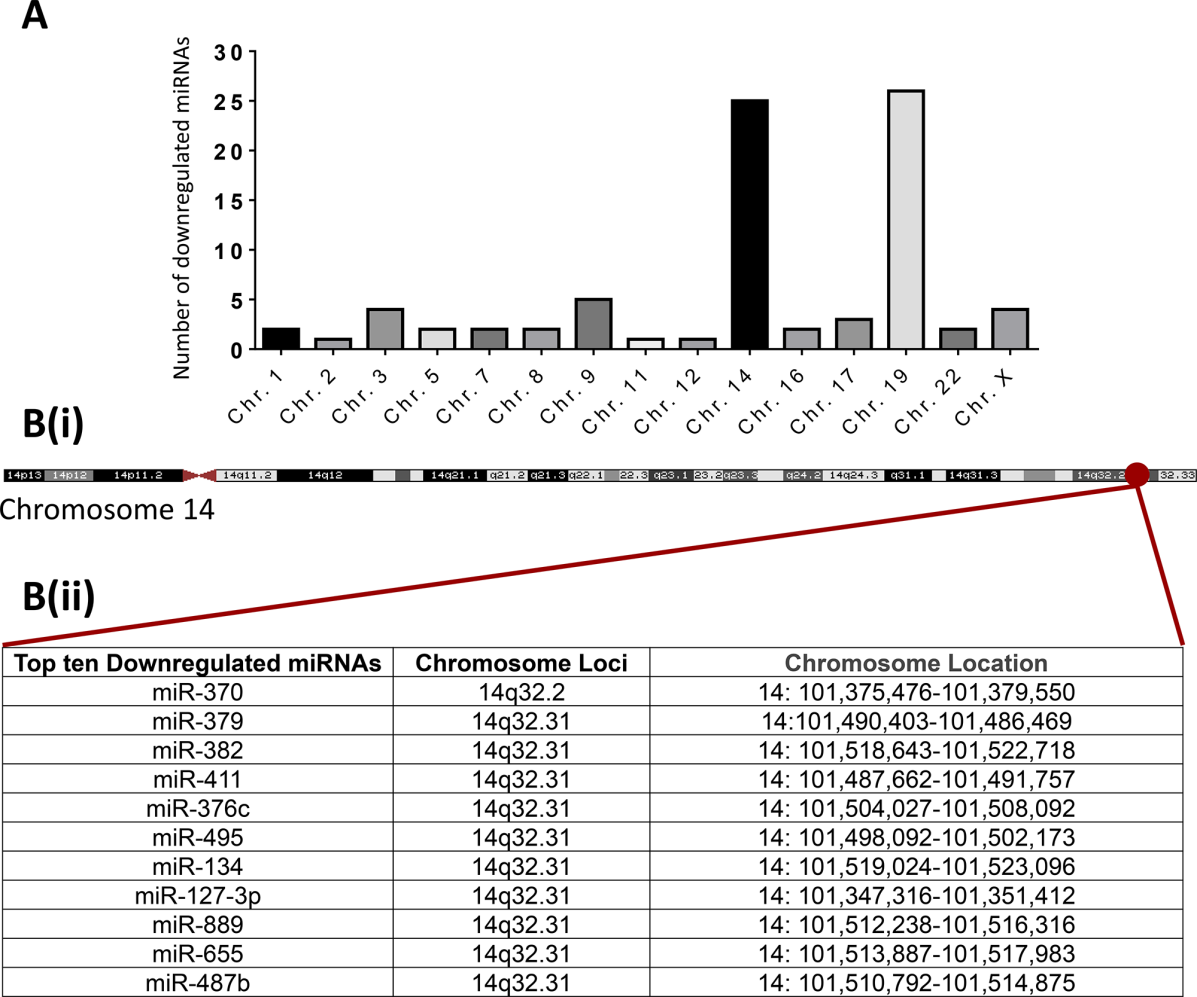
Chromosomal locations of the miRNAs commonly down-regulated in both Hs578Ts(i)_8_ cells and Hs578Ts(i)_8_ EVs The National Center for Biotechnology Information (NCBI) was accessed to identify the chromosomal origin of miRNAs of interest. **A.** Chromosomal origin of the 83 miRNAs down-regulated in Hs578Ts(i)_8_ cells and EVs compared to Hs578T cells and EVs. **B.** Map of chromosome 14, highlighting the locus where all of the ten most substantially down-regulated miRNAs originate.

Investigation of the chromosomal location for the 83 miRNAs commonly down-regulated in Hs578Ts(i)_8_ cells and Hs578Ts(i)_8_ EVs compared to their Hs578T cells and EVs, respectively, indicated that the majority of these miRNAs originate from two chromosomes *i.e.* chromosome 19 (26 miRNAs) and chromosome 14 (25 miRNAs) (Figure 3A). Interestingly, nine of the ten most substantially down-regulated are encoded from 14q32.31, with the tenth miRNA from 14q32.2 (Figure 3B(i) & 3B(ii)). As detailed in Supplementary Material (supported by Suppl. Figures 1, 2, 3, 4, 5), cytogenetic analysis was performed to establish if this region of chromosome 14 was deleted. No structural anomalies at chromosomes 14q32 were observed. However, qPCR assessment for the neighbouring MEG3 locus showed significantly reduced MEG3 expression levels in Hs578Ts(i)_8_ compared to Hs578T cells indicating transcriptional silencing in this chromosomal region in Hs578Ts(i)_8_ cells, reflected in their EV content.

### miR-134 levels are significantly lower in tumour tissue from patients with breast cancer compared to matched normal specimens

**Figure 4:**
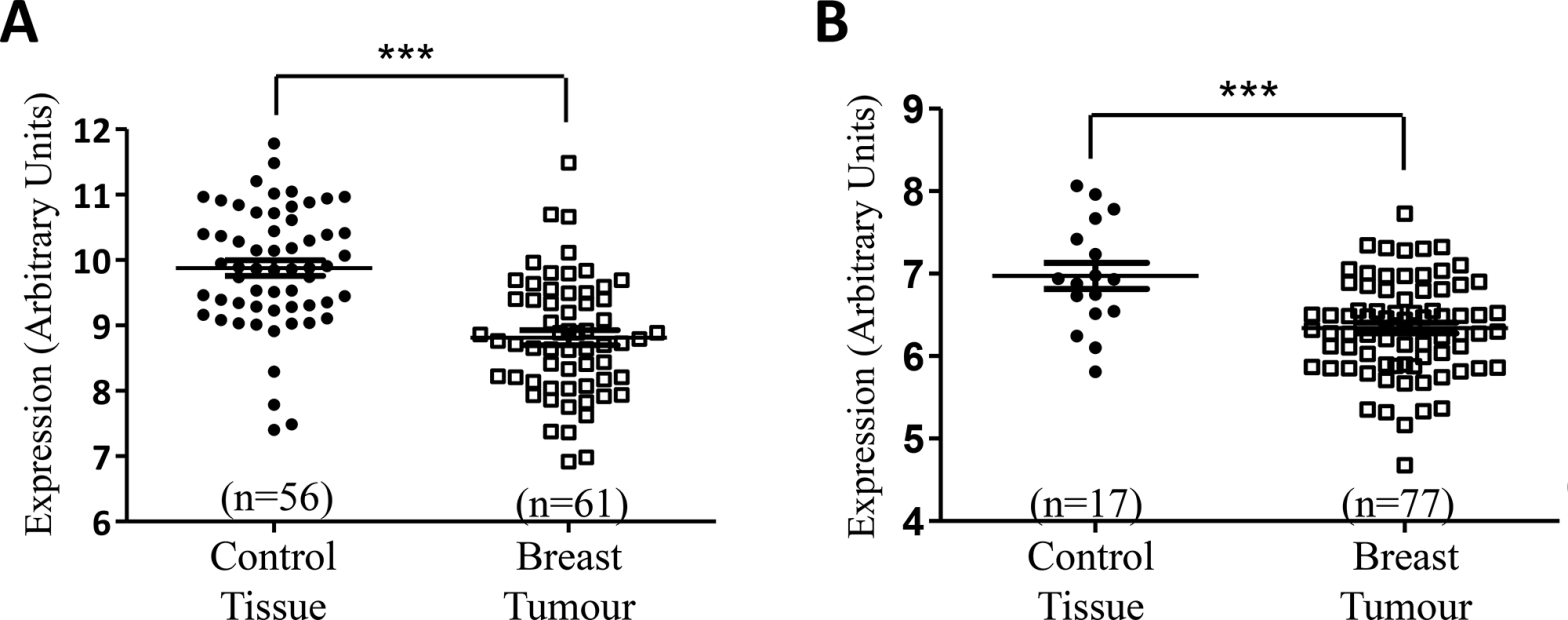
Reduced miR-134 levels are found in breast tumour and serum EV specimens, compared to healthy controls Analysis of the GEO datasets **A.** GSE26659 and **B.** GSE40525 showed that miR-134 is significantly down-regulated in breast tumours when compared to levels in healthy control breast tissue. Graphs are displayed as mean ± SEM, where ***p < 0.001.

As miR-134 was most substantially down-regulated in both Hs578Ts(i)_8_ cells and EVs compared to their Hs578T counterparts, prior to exploring its functional relevance we felt it was important to establish if it may have relevance in breast cancer rather than being solely a cell line related observation. For this we mined relevant publically-available datasets through Gene expression omnibus. From the GSE40525 and GSE26659 datasets, miR-134 was found to be significantly down-regulated in breast cancer (Figure 4A & 4B, respectively) when compared its levels in healthy breast tissue.

### miR-134 over-expression is associated with STAT5B, Hsp90 and Bcl-2 expression and increased cisplatin-induced apoptosis

**Figure 5:**
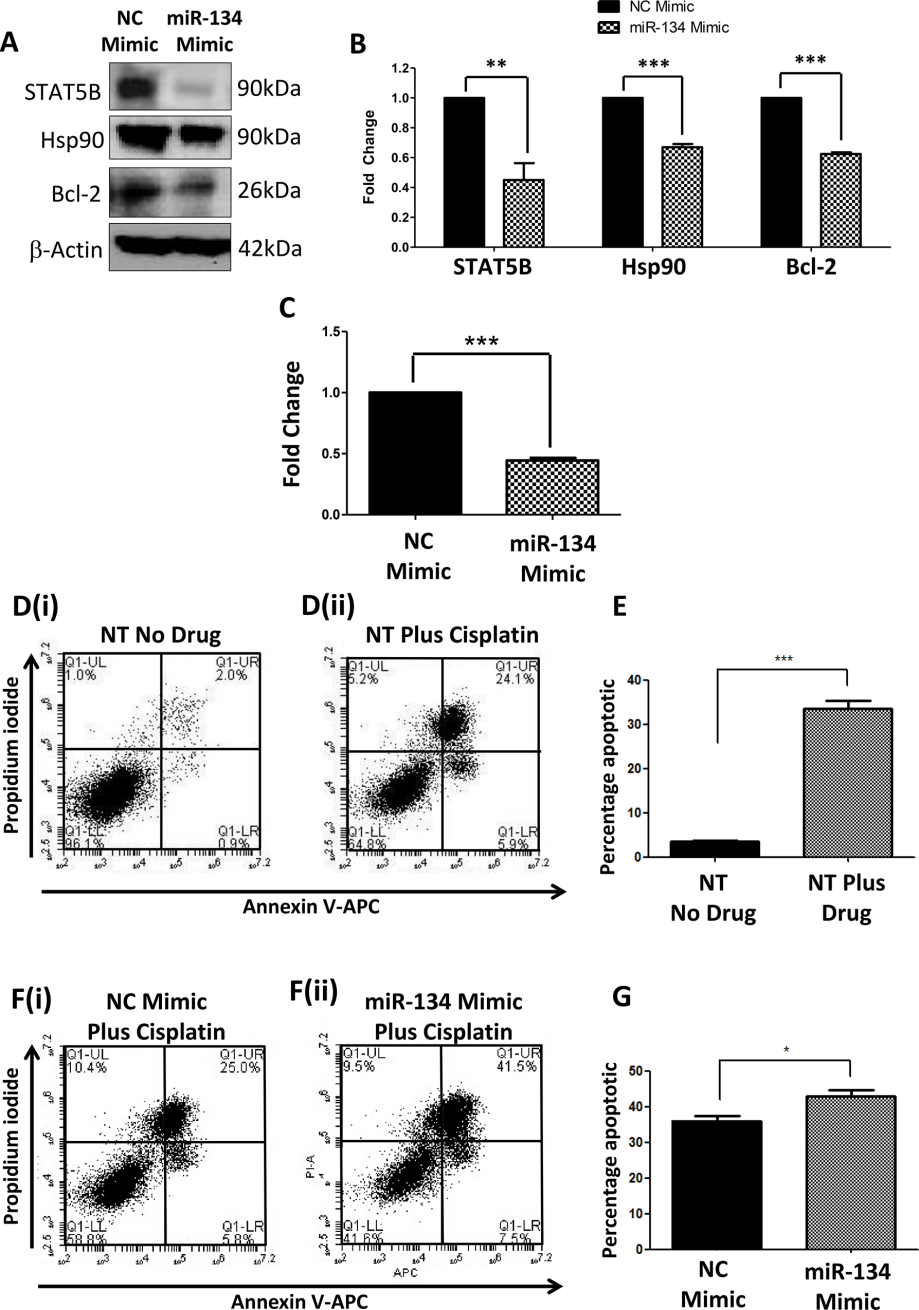
Effects of directly over-expressing miR-134 in Hs578Ts(i)_8_ cells **A.** Immunoblot analysis indicated that STAT5B (predicted target controlled by miR-134), Hsp90 and Bcl-2 are significantly reduced when miR-134 is transfected into Hs578Ts(i)_8_ cells, and **B.** confirmed by densitometry, when compared to the effects of NC-mimic transfection. **C.** Effect of miR-134 transfection compared to NC-mimic transfection on Hs578Ts(i)_8_ proliferation analysed using acid phosphatase assay. **D.** Representative Annexin V-APC and PI scatter plots generated using FACS, for **D(i).** non-transfected (NT) and untreated Hs578Ts(i)_8_ cells, and **D(ii).** non-transfected (NT) Hs578Ts(i)_8_ cells treated with 15 μM cisplatin. **E.** Graphical representation, generated from FACS analysis, of total apoptotic cells detected in non-transfected, untreated cells *versus* non-transfected cells treated with 15 μM cisplatin. **F.** Representative annexin V-APC and PI scatter plots, generated using FACS, for **F(i).** NC-mimic Hs578Ts(i)_8_ cells treated with cisplatin and **F(ii).** miR-134-mimic transfected Hs578Ts(i)_8_ cells treated with cisplatin. **G.** Graphical representation, generated from FACS analysis, of total apoptotic cells detected in NC-mimic *versus* miR-134 transfected cells following treatment with cisplatin. Results are displayed as mean ± SEM, where *p < 0.05; **p < 0.01; ***p < 0.001.

TargetScan predicted miR-134 to target STAT5B at position 2510–2517 3′UTR [25]. In order to assess the functional relevance of miR-134, Hs578Ts(i)_8_ cells were directly transfected with a miR-134-mimic (or negative control (NC)-mimic). Successful transfection and miR-134 over-expression was confirmed by qPCR (Suppl. Figure 6). Immunoblotting showed that STAT5B, Hsp90 and Bcl-2 expressions were significantly reduced by 2.2-fold (*p* = 0.008), 1.5-fold (*p* = 0.0001) and 1.6-fold (*p* = 0.000004), respectively, following miR-134 transfection (Figure 5A & 5B). This was associated with reduced aggressiveness of the Hs578Ts(i)_8_ cells, evidenced by their reduced proliferation (2.2-fold; *p* = 0.00004) (Figure 5C). Migration and invasion were also observed to be reduced (Suppl. Figure 7A & 7B); however this may be due, at least partly, to reduced proliferation. miR-134 over-expression in these Hs578Ts(i)_8_ cells did not significantly augment the anti-proliferative effects of the anti-Hsp90 compounds 17-AAG and PU-H71 (Suppl. Figure 7C).

As ectopic miR-134 over-expression in Hs578Ts(i)_8_ cells was associated with reduced expression of the anti-apoptotic protein Bcl-2, we more extensively analysed the effect of miR-134 transfection on apoptosis in combination with cisplatin treatment. First, we determined the effect of 15 μM cisplatin on non-transfected (NT) cells. After 24 h, flow cytometric analysis showed that 15 μM cisplatin significantly increased the percentage of Hs578Ts(i)_8_ total apoptotic events (early, represented by AnnexinV positive cells, and late, represented by Annexin V and PI positive cells) from 4.6% in untreated cells to 40% in cisplatin-treated cells (*p* = 0.0002) (Figure 5D(i) & 5D(ii) & 5E).

24 h following transfection of Hs578Ts(i)_8_ with miR-134-mimic or NC-mimic, cells were treated with 15 μM cisplatin for 24 h. An additional 10 ± 3% increase (*p* = 0.017) in apoptosis in response to cisplatin in miR-134-mimic *versus* NC-mimic transfected cells was achieved (Figure 5F(i) & 5F(ii) & Figure 5G), again in all cells with characteristics of early and late apoptosis.

### miR-134-enriched EVs released from miR-134-transfected Hs578Ts(i)_8_ cells reduce aggressiveness of secondary cells (apparently via down-regulation of STAT5B-Hsp90) and increased sensitivity to anti-Hsp90 drugs

**Figure 6:**
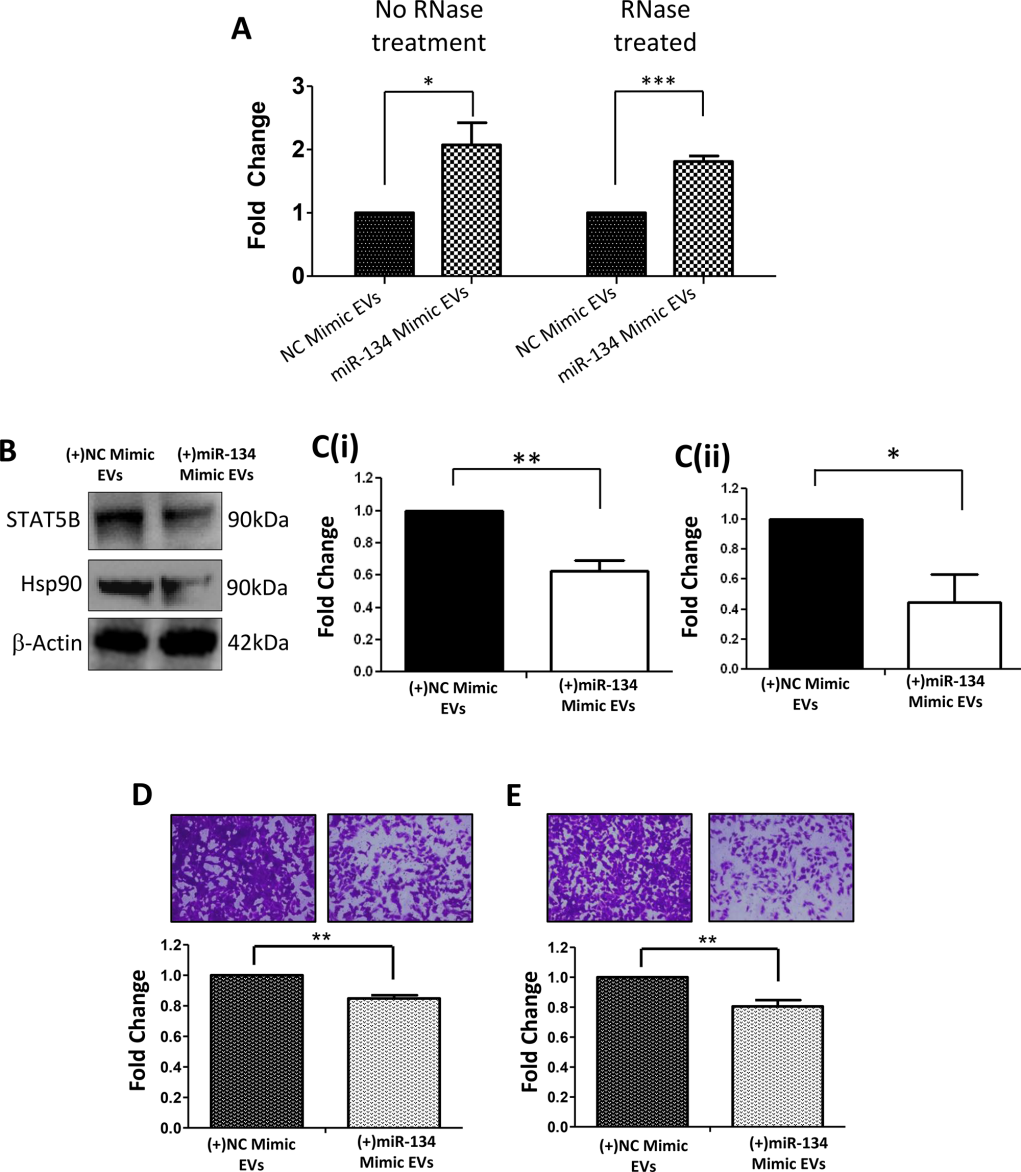
Effects, on recipient cells, of miR-134-enriched EVs expelled from miR-134-over-expressing Hs578Ts(i)_8_ cells **A.** qPCR analysis confirmed that miR-134 is enriched in EVs derived from miR-134-overexpressing Hs578Ts(i)_8_ cells when compared to levels in EVs from NC-mimic-transfected Hs578Ts(i)_8_ cells. **B.** These miR-134-enriched EVs (or NC mimic-EVs) were incubated with Hs578Ts(i)_8_ parent cells resulting in significantly reduced expression of **C(i).** STAT5B and **C(ii).** Hsp90, as determined by immunoblot analysis followed by densitometry. This observation was associated with significantly reduced cellular **D.** migration and **E.** invasion of the recipient parental Hs578Ts(i)_8_ cells. Graphs represent triplicate biological repeats, each including three technical repeats and are displayed as mean ± SEM, where *p < 0.05; **p < 0.01; ***p < 0.001.

Following analysis of directly transfected miR-134 back into Hs578Ts(i)_8_ cells, we proceeded to investigate if EVs from miR-134-transfected Hs578Ts(i)_8_ would be enriched with miR-134 and, if so, could they deliver this miRNA to secondary cells where it could function. Following RNAse-treatment of the EV isolates to ensure that the miR-134 considered was internal to the EVs, qPCR analysis confirmed that EVs expelled from the miR-134-transfected cells were, indeed, enriched with miR-134 (Figure 6A). As a consequence of their subsequent addition to secondary cells (in this case, Hs578Ts(i)_8_ parent cells as they have low endogenous miR-134 levels), STAT5B and Hsp90 protein levels were significantly reduced (1.6-fold, *p* = 0.005; 2.3-fold, *p* = 0.04; Figure 6C(i) & 6C(ii), respectively). Furthermore, a significant reduction of Hs578Ts(i)_8_ migration (1.2-fold; *p* = 0.002; Figure 6D) and invasion (Figure 6E; 1.2-fold; *p* = 0.009) resulted. Here, unlike directly transfecting miR-134, the application of miR-134-enriched EVs did not significantly alter Hs578Ts(i)_8_ proliferation or cisplatin-induced apoptosis (Suppl. Figure 8).

As treatment with miR-134-enriched EVs has the ability to reduce Hsp90 expression levels and did not impact on cellular proliferation, we examined if miR-134-enriched EVs could increase sensitivity to anti-Hsp90 drugs. Here, miR-134-enriched EVs increased the sensitivity of Hs578Ts(i)_8_ cells to their approximate IC_50_ concentration of 17-AAG and PU-H71; drugs that robust pre-clinical studies have indicated to possess potential as anti-cancer treatments in TNBC and thus have been proposed as relevant drugs to advance to clinical trials in TNBC [7, 27].

**Figure 7:**
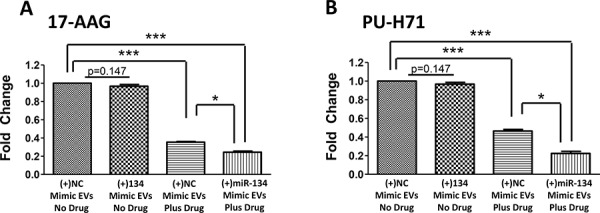
Treating Hs578Ts(i)_8_ cells with miR-134-enriched EVs substantially increases the anti-proliferative effects of anti-Hsp90 compounds, 17-AAG and PU-H71 **A.** Treating Hs578Ts(i)_8_ cells with miR-134-enriched EVs increases cellular sensitivity to 17-AAG compared to the effects of NC-mimic EVs, analysed using acid phosphatase assay. **B.** Similarly, treating Hs578Ts(i)_8_ cells with miR-134-enriched EVs increases cellular sensitivity to PU-H71 compared to the effects of NC-mimic EVs, determined using acid phosphatase assay. Graphs represent triplicate biological repeats, each including three technical repeats, displayed as *n* = 3 mean ± SEM, where *p < 0.05, **p < 0.01, ***p < 0.001.

Regarding 17-AAG (Figure 7A), cells treated with miR-134-enriched EVs showed 4.1-fold reduced growth (*p* = 0.0000004) in response to 17-AAG, compared to cells treated with NC mimic-enriched EVs and no drug. This is in comparison to 2.8-fold increased toxicity (*p* = 0.00000007) with cells treated with NC-mimic EVs and 70 nM 17-AAG. Comparing the effects of NC-mimic EVs to miR-134-mimic EVs, the latter produced a 1.5-fold (*p* = 0.001) increase in sensitivity to 17-AAG (Figure 7A).

Likewise with PU-H71 (Figure 7B), cells treated with miR-134-enriched EVs showed 4.5-fold toxicity (*p* = 0.000004) in response to PU-H71, compared to cells treated with NC-mimic-enriched EVs and no drug. This is in comparison to 2.2-fold increase in toxicity (*p* = 0.000007) with NC-mimic EVs and PU-H71. Comparing the effects of miR-134-mimic EVs and NC-mimic EVs, a 2.1-fold (*p* = 0.0001) increase in Hs578Ts(i)_8_ sensitivity to PU-H71 was observed (Figure 7B).

## DISCUSSION

Advancing on our previous analysis of exosomes in relation to TNBC [23], we believe this to be the first study to profile the miRNA contents of both TNBC derived-EVs and cells to identify miRNAs which may have therapeutic potential in TNBC and aim to exploit these EVs as therapeutic miRNA delivery vesicles to reduce TNBC aggression and increase TNBC drug sensitivity. Additionally, this is the first report detailing the relevance of miR-134 in TNBC, as well as its potential as a tumour suppressor.

Here, we observed that the miRNA contents of EVs are highly representative of the miRNAs contents of their cells of origin, an observation made by ourselves and others in other cancer types including melanoma [28], ovarian [29], oesophageal [30] and prostate [31] cancers. Of particular interest to identifying a tumour suppressor miRNA, we found 83 miRNAs commonly down in both Hs578Ts(i)_8_ cells and their EVs compared to Hs578T counterparts. It has been established that human miRNAs tend to be derived from fragile chromosomal regions or genomic regions associated with cancer progression [32]. Notably, the ten most substantially down-regulated miRNAs in Hs578Ts(i)_8_ cells/EVs all stem from the same chromosomal cluster of chromosome 14, 14q32, which has been identified as a region commonly deleted in cancer progression [33–38]. Additionally, down-regulated miRNAs derived from this cluster have been suggested to be involved in cancer progression [39–42]. Noncoding genes at this locus, including miR-134 and the co-regulated MEG3, are physiologically expressed from the maternal allele [43]. Our cytogenetic analysis detected no structural anomalies at chromosomes 14q32, although significantly reduced expression of neighbouring “sentinel” MEG3 locus was observed in Hs578Ts(i)_8_ compared to Hs578T cells, suggesting transcriptional silencing in this chromosomal region of Hs578Ts(i)_8_ cells; which is reflected in their EV content.

miR-134 (from 14q32.31) showed the greatest level of reduction in the Hs578Ts(i)_8_-derived EVs. This suggests that loss, or at least reduced levels, of miR-134 may play an important role in TNBC aggressiveness. Similar observations to this have recently been reported in other cancers where miR-134 levels were found to inversely correlate with the aggression of NSCLC [44] and osteosarcoma [45]. Additionally, miR-134 exerts anti-tumour effects in NSCLC, as well as playing a tumour suppressive role in hepatocellular carcinoma [46]. However, no such observation has been reported in breast cancer to the best of our knowledge. Strengthening our observations, our analysis of two independent publically-available datasets demonstrated that miR-134 levels are significantly reduced in breast cancer tumours compared to normal control tissue. Similar to other miRNA profiling studies performed in breast cancer, here we also identified miR-31 and miR-245 to be down-regulated in our aggressive TNBC cells and EV variants. Although we did not find miR-15a, miR-126 and miR-200b to be down-regulated in our Hs578Ts(i)_8_ cells, we identified them to be present at reduced levels in the corresponding EVs, in keeping with observations made by others in breast cancer [18]. Complimenting others [47], we found that miR-200a is down-regulated in more aggressive breast cancer cells [47]. However, neither of these studies [18, 47] identified miR-134 as being reduced in breast cancer.

We, therefore, analysed the functional relevance of miR-134 in the aggressive TNBC cell line, Hs578Ts(i)_8_. We established that directly re-introducing/transfecting miR-134 back into these cells, from where it has been substantially lost, resulted in significantly reduced levels of its predicted target protein, STAT5B. Of particular interest, it has been previously reported that STAT5B induces the transcription of Hsp90, a molecular chaperone protein widely expressed in breast cancer [26]. Hsp90 is implicated in increasing the survival of breast cancer cells by stabilising oncogenic proteins [48] including Bcl-2, and if targeted, can allow tumour cells to overcome apoptotic resistance through Hsp90-Bcl-2 interactions [49]. As mentioned above, Hsp90 is also a potential therapeutic target for TNBC [7]. Here its expression was significantly reduced as a consequence of miR-134 over-expression.

As Bcl-2 expression was also observed to be decreased upon miR-134 transfection, we initially opted to investigate miR-134′s potential relevance as an apoptosis inducer/enhancer. Transfection of Hs578Ts(i)_8_ cells with miR-134-mimic in combination with cisplatin treatment [50] increased cellular sensitivity to cisplatin-induced apoptosis. This result suggests that miR-134 may have potential as an onco-suppressor when used in combination with chemotherapeutics such as cisplatin. This finding compliments previous observations that miR-195, miR-24-2 and miR-365-2 amplify the apoptotic effect of etoposide in MCF7 breast cancer cells [51]. Further investigations of the effects of miR-134 in TNBC aggression led us to analysing its effects on migration and invasion. Although miR-134 appears to significantly reduce the invasion and migration of TNBC cells, it apparently does not give substantial benefit to migration and invasion additional to its effects on proliferation (which would cause fewer cells to be available to migrate or invade and thus give the, possibly misleading, impression of reduced migration and invasion). Furthermore, combining the direct transfection of miR-134-mimic with Hsp90 inhibitors 17-AAG or PU-H71 treatment [7] did not significantly amplify the anti-proliferative effects of 17-AAG or PU-H71 on Hs578Ts(i)_8_ cells. Together, these results show that directly transfecting miR-134 back into Hs578Ts(i)_8_ cells resulted in significantly reduced cellular growth rate and reduced levels of STAT5B and subsequently Hsp90, but with no obvious effects on migration/invasion or response to anti-Hsp90 agents additional to its effects on growth rate. This direct transfection of miR-134 did, however, significantly enhance the apoptotic response to cisplatin.

As EVs are often proposed as having potential as “self” delivery systems [52, 53] we subsequently investigated here if EVs could be manipulated as miR-134 delivery vesicles. Here, post-transfection of Hs578Ts(i)_8_ cells with miR-134, the released miR-134-enriched EVs resulted in down-regulation of both STAT5B and Hsp90 in recipient (Hs578Ts(i)_8_) cells and they significantly increased sensitivity to anti-Hsp90 inhibitors, 17-AAG and PH-H71. Furthermore, migration and invasion of the recipient cells was significantly reduced, without effect on proliferation or cellular response to cisplatin.

Overall, here we showed that the miRNA content of EVs is highly representative of their cells of origin. The miRNAs most substantially down-regulated in the aggressive TNBC cells and their EVs originate from 14q32, a region commonly deleted in cancer progression. Analysis of miR-134 *i.e*. the most substantially down-regulated miRNA, supported its clinical relevance in breast tumours. miR-134 was predicted to control STAT5B expression and our functional studies support its effects to be, at least partly, *via* this route. Delivery of miR-134 into Hs578Ts(i)_8_ cells substantially changed their phenotype. Specifically, when delivered directly by transfection the STAT5B and Hsp90 expression levels were reduced, but response to anti-Hsp90 drugs was not augmented. However, cellular growth was reduced and cisplatin-induced apoptosis was enhanced. Delivery *via* miR-134-enriched EVs also reduced STAT5B and Hsp90 expression, had no apparent effects on proliferation, but cellular migration and invasion were reduced and sensitivity to anti-Hsp90 drugs was enhanced. While the phenotypic influences observed are likely to be, at least partly, due to the amounts of miR-134 delivered (∼15,000-fold by transfection *versus* < 2-fold *via* EVs), these studies-originating with EV analysis- have identified miRNA-134 as a potential biomarker and therapeutic for breast cancer. Future studies aimed at titrating the amount of miR-134 delivered may help to achieve maximum benefit. Of course a limitation of this study is that fact that we only had access to one pair of isogenic TNBC cell line variants. Expanding this work to a more extensive analysis of a larger number of cell lines (and their isogenic variants, if possible) would be ideal and is recommended.

In conclusion, these studies indicate the potential diagnostic relevance of miR-134 both as a biomarker for TNBC and as a potential therapeutic option. In addition to extending these studies to more cell line models and analysis of independent cohorts of breast tumour and normal tissue specimens, investigating the prognostic and predictive nature of miR-134 would be of value. We propose that such studies could initially include analysis of specimens procured retrospectively where patients’ response to systemic treatment (*e.g*. cisplatin and/or Hsp90 inhibitors) and/or survival outcome is now already known. Positive results emerging from such analysis could then be advanced to blinded prospective longitudinal studies, statistically powered based on the outcome from the retrospective analysis, where specimens would be procured prior to any treatment, during the course of treatment and post-treatment to help determine and validate the true relevance of miR-134 in TNBC.

## MATERIALS AND METHODS

### Cell culture

Hs578T (ATCC, Manassas, VA, USA), a TNBC cell line, and its isogenic sub-clone Hs578Ts(i)_8_ cells (gift from Dr. Linda Hughes and Dr. Susan McDonnell [24]) were cultured at 37°C/5% CO_2_ in DMEM (Sigma-Aldrich, St. Louis, MO, USA)), 10% FBS (PAA, Pasching, Austria), 2 mM L-Glutamine (Sigma-Aldrich), and 10 μg/ml of insulin (Sigma-Aldrich). Cell line authentication is described in the Supplementary Material.

### EVs isolation

#### EVs from Hs578T and Hs578Ts(i)_8_ conditioned medium

EVs were isolated from Hs578T and Hs578Ts(i)_8_ conditioned medium (CM) by filtration and ultracentrifugation and were quantified as we previously described [23].

#### EVs from miR-134 transfected cells

Hs578Ts(i)_8_ cells were transfected with miR-134 (as described below) in medium containing EV-depleted FBS (dFBS) [23] and cultured for 48 h. 10 ml of CM was collected and treated with 5 μg/ml RNase A (Sigma-Aldrich) for 30 mins at 37°C, according to a previous study [22]; centrifuged at 3 000 *g* for 15 min to remove cellular debris. EVs were then isolated from the supernatants using Exoquick (System Biosciences, Mountain View, CA, USA) and re-suspended in 100 μl of PBS, following manufacturer's instructions and as previously described [22].

### Immunoblotting

For immunoblotting, cell pellets and EVs were lysed using SDS lysis buffer (250 nM Tris-HCL, pH 7.4; 2.5% SDS). Proteins (30 μg) were resolved on 12.5% SDS gels (Lonza, Basel, Switzerland) and transferred onto PVDF membranes (Bio-Rad). Blots were blocked in 5% (w/v) BSA in PBS containing 0.1% Tween-20 and incubated overnight at 4°C with primary antibodies to PDC6I/Alix (1:1000; Abcam, Cambridge, UK), TSG101 (1:1000; Abcam), CD63 (1:500; Abcam), STAT5B (1:500; Cell Signalling, Danvers, MA, USA), total Hsp90 (1:1000; StressMarq, Victoria, Canada), Bcl-2 (1:500; Calbiochem, San Diego, USA) or β-Actin (1:1000, Sigma-Aldrich). Secondary antibodies were incubated for 1 h at room temperature. Blots were developed as we previously described [23].

### Transmission electron microscopy

For transmission electron microscopy (TEM), approximately 10 μl aliquot of EVs were examined at 100 kV using a JEOL JEM-2100 TEM (JOEL, Peabody, USA), as we previously described [23].

### RNA isolation

Total RNA was isolated from cells and EVs using the miRNeasy mini kit (Qiagen, Venlo, The Netherlands) following manufacturer's instructions. RNA was quantified by Nanodrop-1000 spectrophotometer (Thermo Scientific, Wilmington USA).

### miRNA profiling

miRNA profiling (384 miRNAs) was performed on biological triplicates of Hs578T and Hs578Ts(i)_8_ cells and their derived EVs using TaqMan Low-Density Assays (TLDA). qPCR reactions were performed according to the manufacturer's instructions (Applied Biosystems, Foster City, CA, USA). Briefly, 20 ng of RNA was reverse transcribed using TaqMan miRNA RT Kit in combination with the Megaplex primers pool set A. The resulting cDNA was pre-amplified using the TaqMan PreAmp Master Mix and Megaplex PreAmp primers. TLDAs were run using a ViiA7 Real-Time PCR System (Applied Systems). Cycle threshold values were calculated using SDS software. C_T_ values ≥35 were considered as undetected [54]. Relative quantities of miRNAs were calculated using the 2^−ΔΔ*C*T^ method after normalisation to RNU48 as control, which was established as unchanged between Hs578T and Hs578Ts(i)_8_ cell and EV samples. A ≥2-fold change and *p* < 0.05 were cut-offs for determining if a miRNA was comparatively up- or down-regulated.

### qPCR

In order to validate key findings from the miRNA profiling, qPCR was performed for specific miRNAs of interest. All TaqMan miRNA assays used (miR-134: ID:001186; miR-370: ID:002275; miR-655: ID:001612; miR-376c: ID: 002122; miR-889: ID:002202 and miR-146b-3p: ID:002361) were from Applied Biosystems. cDNA synthesis was performed using TaqMan miRNA Reverse Transcription assays kit (Applied Biosystems) according to manufacturer's instructions. Briefly, reverse transcriptions were performed on biological triplicates, using 10 ng total RNA and qPCR was subsequently performed using TaqMan microRNA assay kit (Applied Biosystems) using 1.33 μl of cDNA from reverse transcription. Relative quantities of miRNA were calculated using the 2^−ΔΔ*C*T^ method for cells and EVs after normalisation to RNU48.

### miR-134 in breast tumour and normal tissue specimens

miR-134 levels in breast tumours and matched normal breast tissue were determined using two publically-available tumour datasets by Gene expression omnibus (GEO) (http://www.ncbi.nlm.nih.gov/geo/). Specifically GEO accession GSE26659 [55] is comprised of a total of 77 breast tumour specimens obtained from patients who underwent primary surgical treatment and 17 normal breast tissue specimens obtained from mammoplastic reductions. GEO accession GSE40525 [56] consists of breast tumour specimens (*n* = 61) with 56 control tissues obtained from matched adjacent peri-tumoural normal breast tissues. miRNA expression values were determined using GEO2R [57].

### Transfection of miR-134 into Hs578Ts(i)_8_ cells

Hs578Ts(i)_8_ cells were transfected with miR-134-mimic (4464066; Applied Biosystems) or NC-mimic (4464058; Applied Biosystems) at a final concentration of 30 nM, as per manufacturer's instructions. Briefly, Hs578Ts(i)_8_ cells were seeded at 1.5 × 10^5^ cells/well (6-well plate). The following day, medium was replaced with fresh complete medium. 5 μl of lipofectamine 2000 (Invitrogen, Carlsbad, CA, USA) per well was diluted in 250 μl Opti-MEM medium (Invitrogen). miRNA-mimics were diluted in 250 μl Opti-MEM and incubated for 5 min. The lipofectamine/Opti-MEM mixture was subsequently mixed with the miRNA/Opti-MEM mixture and incubated for 20 min at room temperature. This was subsequently added to the cells and allowed to transfect for 4 h at 37°C/5% CO_2_. Medium was then removed and cells were gently washed twice with base medium and fed with complete medium.

### Growth rates following miR-134 re-introduction into Hs578Ts(i)_8_ cells

#### With direct miR-134 transfection

Proliferation analysis was initiated 48 h post transfection of cells and cultured for 48 h before analysing growth. Specifically, transfected Hs578Ts(i)_8_ cells (with miR-134-mimic or NC-mimic) were seeded at 2 × 10^3^ cells/well in a 96-well plate and cultured for 48 h, when growth was assessed using the acid phosphatase assay [58].

#### With miR-134-enriched EVs

Hs578Ts(i)_8_ cells were seeded at a density of 2 × 10^3^ cells/well in a 96-well plate in the presence of 2 μg of EVs from miR-134-mimic or NC-mimic transfected cells. After 48 h, growth was assessed using acid phosphatase^51^.

### Effect of miR-134 on cisplatin-induced apoptosis

#### With direct miR-134 transfection

To analyse the effects of miR-134 on apoptosis induction, cells were transfected with miR-134-mimic or NC-mimic and, 24 h later, transfected cells were treated with 15 μM cisplatin for a further 24 h. CM was then collected and cells were trypsinised and pelleted. CM was used to neutralise trypsin and to collect any apoptotic cells which may be present in the CM. Cell pellets were re-suspended in 2 ml 1X binding buffer (BB) (0.1 M HEPES, 1.4 M NaCl, 25 mM CaCl_2_, pH 7.4), centrifuged at 200 g and supernatant discarded and resuspended in 30 μl of BB solution. 20 μl of cell suspension, 5 μl of Annexin-V-allophycocyanin (APC) (BD Biosciences) and 5 μl of propidium iodide (PI) staining solution (BD Biosciences) were mixed. 70 μl of 1X BB was added and incubated at room temperature in the dark for 15 mins. 400 μl of 1X BB solution was subsequently added and mixed by pipetting. Levels of apoptosis was analysed on 2 × 10^4^ cells using a BD Accuri™-C6 flow cytometer.

#### With miR-134-enriched EVs

Hs578Ts(i)_8_ cells were seeded at 1.5 × 10^5^ cells/well in a 6-well plate in the presence of 5 μg of miR-134-mimicEVs or NC-mimic EVs and cultured for 24 h. Medium was then removed and cells were treated with 15 μM of cisplatin and re-treated with 5 μg of the same EVs. 24 h later, cells were analysed for apoptosis as above.

### Migration and invasion following miR-134 re-introduction into Hs578Ts(i)_8_ cells

#### With direct miR-134 transfection

Migration and invasion assays were performed as we previously described [23, 59]. 48 h post-transfection of Hs578Ts(i)_8_ cells with miR-134-mimic. Briefly, medium (500 μl) containing 10% dFBS was added to each well, below the insert. 2.5 × 10^4^ cells in 500 μl complete medium were added to each insert (for migration assay, cells were seeded on a non-coated transwell chamber, whereas for invasion, cells were seeded on an ECM-coated transwell chamber). Five hours later, when cells had attached, medium within the inserts was replaced with medium containing only 1% FBS. After allowing 48 h for migration, cells were stained and evaluated [23].

#### With miR-134-enriched EVs

Hs578Ts(i)_8_ cells were seeded at 2.5 × 10^4^ cells on an uncoated 8 μm pore sized 24-well transwell chamber for migration and an ECM-coated transwell chamber for invasion and allowed to attach for 5 h in complete medium. This was then replaced with DMEM including 1% FBS and 10 μg (migration) or 15 μg (invasion) of miR-134-mimic or NC-mimic EVs were added. Cells were cultured for 48 h and analysed as above.

### Treating Hs578Ts(i)_8_ cells for immunoblotting

Hs578Ts(i)_8_ cells were seeded at 2 × 10^4^ cells/well in 6-well plates 40 μg of EVs from miR-134-mimic or NC-mimic transfected cells were added and cultured for 48 h. Cells were prepared for immunoblotting as described above.

### Effects of miR-134 re-introduction on response to anti-Hsp90 drugs

#### With direct miR-134 transfection

To evaluate the effects of Hsp90 inhibitors on Hs578Ts(i)_8_ cells following direct over-expression of miR-134, 48 h post-transfection with miR-134-mimic or NC-mimic cells were seeded at 2 × 10^3^ cells/well into a 96-well plate in 100 μl of complete medium. 24 h later, cells were exposed to their ∼IC_50_ of 17-(Allylamino)- 17-demethoxygeldanamycin/17-AAG (70 nM; Trade name: Tanespimycin) (Sigma-Aldrich) or PU-H71 (60 nM) (Sequoia, Pangbourne UK) drugs. Cell viability was assessed, 48 h later, using acid phosphatase [58].

#### With miR-134-enriched EVs

To investigate the effects of Hsp90 inhibitors on Hs578Ts(i)_8_ cells following exposure to miR-134-mimic EVs or NC-mimic EVs, Hs578Ts(i)_8_ cells were seeded at 2 × 10^3^ cells/well in a 96-well plate. The following day, EVs (2 μg) derived from miR-134-mimic or NC-mimic-transfected cells were added with 17-AAG or PU-H71 drugs and analysed as above [58].

### Statistical and bioinformatics analysis

Online miRNA target prediction software (TargetScan Human Release 6.2) predicted protein targets regulated by miR-134. Chromosomal origins of miRNAs were identified using miRBase. Statistical analysis was performed in Excel. *P*-values were generated using Student's *t*-tests, with *p* < 0.05 considered significant. GraphPad Prism 5.0 was used for graph generation (GraphPad Software Inc, La Jolla, USA).

### Supplementary information

Supplementary information accompanies the paper.

## SUPPLEMENTARY MATERIAL


