# Supplementary material for: miR-134 in extracellular vesicles reduces triple-negative breast cancer aggression and increases drug sensitivity
Source: Oncotarget. 2015 Sep 24;6(32):32774–89. doi: 10.18632/oncotarget.5192 (PMC4741729; doi:10.18632/oncotarget.5192)
Supplement: Supplementary file 1 [file oncotarget-06-32774-s001.pdf]

SUPPLEMENTARY MATERIAL

Cytogenetic analysis of a region of chromosome 14 (chromosome 14q32) to detected possible deletions or rearrangements

METHODS

Genomic DNA from 3–5 × 10^6 cells was prepared from Hs578T and Hs578Ts$(i)_8$ cells using the High Pure PCR Template Preparation Kit (Roche, Basel, Switzerland) according to the manufacturer's instructions, analysed by DNA short tandem repeat profiling and identity verified with reference to ATCC profiles via the DSMZ terminal of the joint cell bank interrogative online database [1]. Cultured cells from each cell line variant were harvested using standard cytogenetic procedures, notably fluorescence *in situ* hybridization (FISH) using bacterial artificial chromosome (BAC) and fosmid probes [2–3]. BAC DNA was prepared using the Big BAC DNA isolation kit (Princeton Separations, Freehold Township, NJ/USA) following the manufacturer's protocol. Circa 1–2 μg DNA was labelled by nick translation (Life Technologies, Darmstadt/Germany) using contrastingly labelled dUTP-fluors (Dy495, Dy590, Dy547) purchased from Dyomics (Jena/Germany). FISH preparations were counterstained with DAPI (4′,6-Diamidine-2′-phenylindole dihydrochloride) obtained from Sigma-Aldrich (Taufkirchen/Germany). Microscopy was performed using a Zeiss Axioimage (Jena/Germany) configured to a HiSKY imaging system (Applied Spectral Imaging, Neckarhausen/Germany). The mapping coordinates of these probes are shown on Suppl. Figure 1 together with their labelling colours and regions of interest (ROI-1/2) covering the miR clusters. The maximum resolution of these methods is circa +/−20Kbp. To extend resolution to the genomic regions of both ROIs, FISH was then performed in Hs578Ts$(i)_8$ using shorter (~40 Kbp) formid clones.

qPCR for DLK1 and MEG3 expression was performed using TaqMan assay (MEG3, ID:4448892; DLK1, ID: 4453320, Applied Biosciences). In brief, RNA was extracted using TriReagent™ as per manufacturer's instructions. Reverse transcription was carried out using 500ng of cellular RNA using 500 ng/1 oligo dT primers (Eurofins MWG Operon), dNTP, MMLV-RT, RNasin and 10X buffer (All Sigma-Aldrich). $2^{-\Delta\Delta CT}$ method was used to calculate the expression levels of MEG3 and DLK1 after normalisation to GAPDH (ID: 4331182) as control, which was unchanged between Hs578T and Hs578Ts$(i)_8$.

RESULTS

Standard chromosome painting revealed that Hs578T is biclonal triploid/hexaploid (Suppl. Figure 2) as previously described by ATCC. No structural rearrangements affecting chromosome 14q32 were detected. Hs578Ts$(i)_8$ was found to be quasi-diploid, carrying one normal and one rearranged copy of chromosome 14. The rearrangement involved duplication of a small amount of unidentified material deriving from chromosome 14 present on the short arm. FISH using flanking - respectively, RP11–90g22 (centromeric) and 168l7 (telomeric) and straddling (RP11–9D19) - BAC clones showed normal configurations in both Hs578T and Hs578Ts$(i)_8$, and in the latter excluded involvement of the ROIs in the chromosome rearrangement present in this cell line (Suppl. Figure 3). FISH using fosmid clones confirmed the results using BAC clones, namely that the ROIs in Hs578Ts$(i)_8$ have maintained a wild type configuration (Suppl. Figure 4).

Cytogenetic analysis detected no anomalies at the ROIs at chromosome 14q32.2. Hence the anomalous micro-RNA silencing observed in this region is not attributable to structural cytogenetic rearrangement, such as micro-deletion or translocation.

It is possible, however, that loss of a whole copy of chromosome 14 in the Hs578Ts$(i)_8$ variant was accompanied by transcriptional silencing, given that genes in this region, including those within ROI-1/2, are normally expressed from the maternal homolog [4–5]. If this mechanism underlies transcriptional silencing, it follows that both remaining copies of chromosome 14 in Hs578Ts$(i)_8$ are paternal in origin. This explanation offers a couple of predictions, namely that genes on chromosome 14 should be homozygotic and that coordinately expressed non-protein coding genes, notably MEG3, in the neighbouring DLK1/MEG3 loci should also be silent. While DLK1 was undetected in both cell line variants, MEG3 was detected in Hs578T cells but its expression was significantly decreased in the Hs578Ts$(i)_8$ variant, supporting the proposal of transcriptional silencing of this chromosomal region due to uniparental disomy (Suppl. Figure 5).

REFERENCES

1. Dirks WG, Drexler HG. STR DNA typing of human cell lines: detection of intra- and interspecies cross-contamination. Methods Mol Biol. 2013; 946:27–38.

2. MacLeod RA, Kaufmann M, Drexler HG. Cytogenetic harvesting of commonly used tumor cell lines. Nat Protoc. 2007; 2:372–382.
3. MacLeod RA, Drexler HG. Classical and molecular cytogenetic analysis. Methods Mol Biol. 2013; 946:39–60.
4. Edwards CA, Mungall AJ, Matthews L, Ryder E, Gray DJ, Pask AJ, Shaw G, Graves JA, Rogers J, Dunham I, Renfree MB, Ferguson-Smith AC. The evolution of the DLKI-DIO3 imprinted domain in mammals. PLoS Biol. 2008; 6:e135.
5. Hagan JP, O'Neill BL, Stewart CL, Kozlov SV, Croce CM. At least ten genes define the imprinted Dlk1-Dio3 cluster on mouse chromosome 12qF1. PLoS One. 2009; 4:e4352.

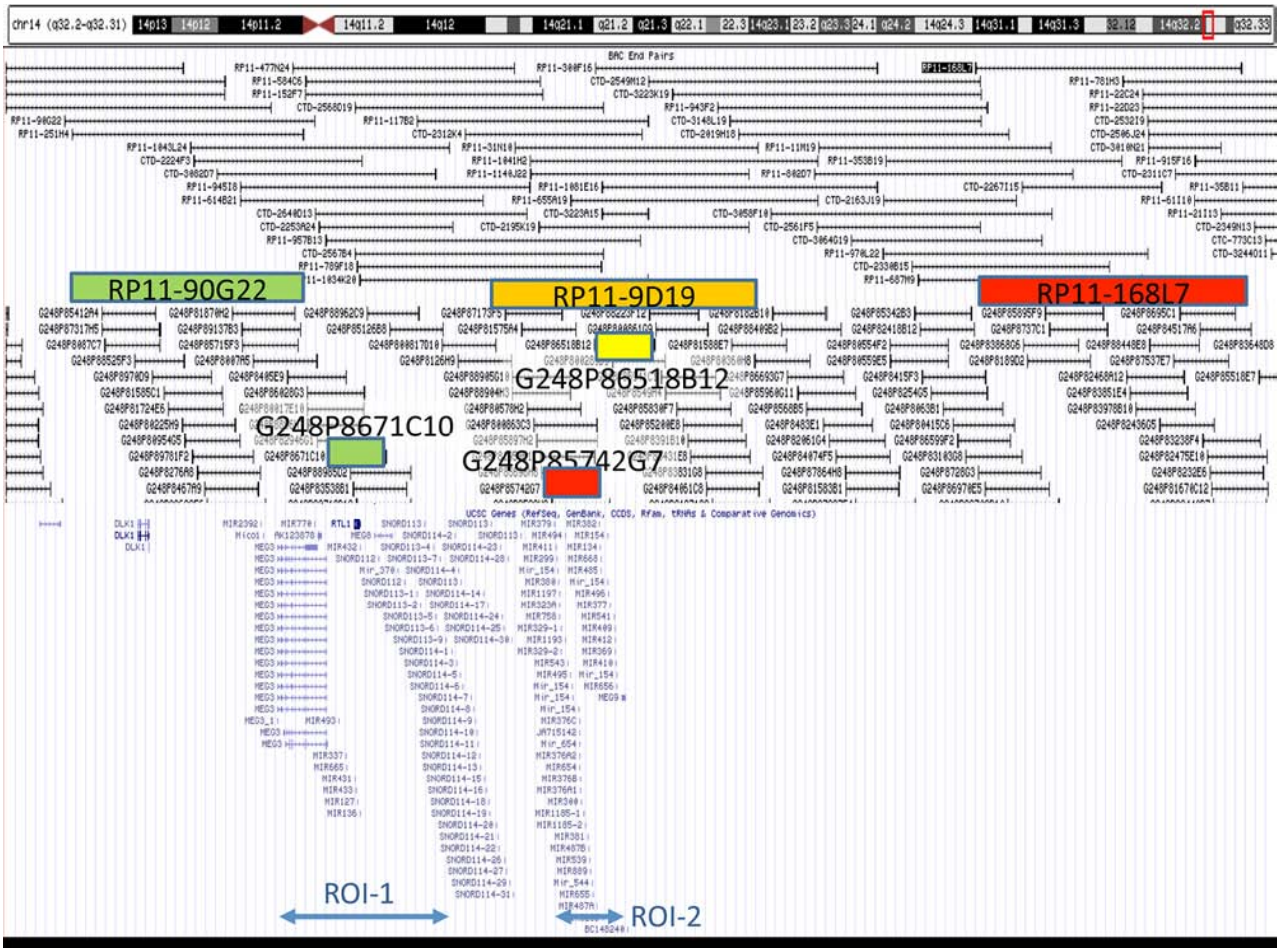

Supplementary Figure S1: Genomic map of part of chromosome 14q32.2 showing clone coordinates, ROIs and BAC and fosmid clone coordinates used in this study, together with the labelling scheme used.

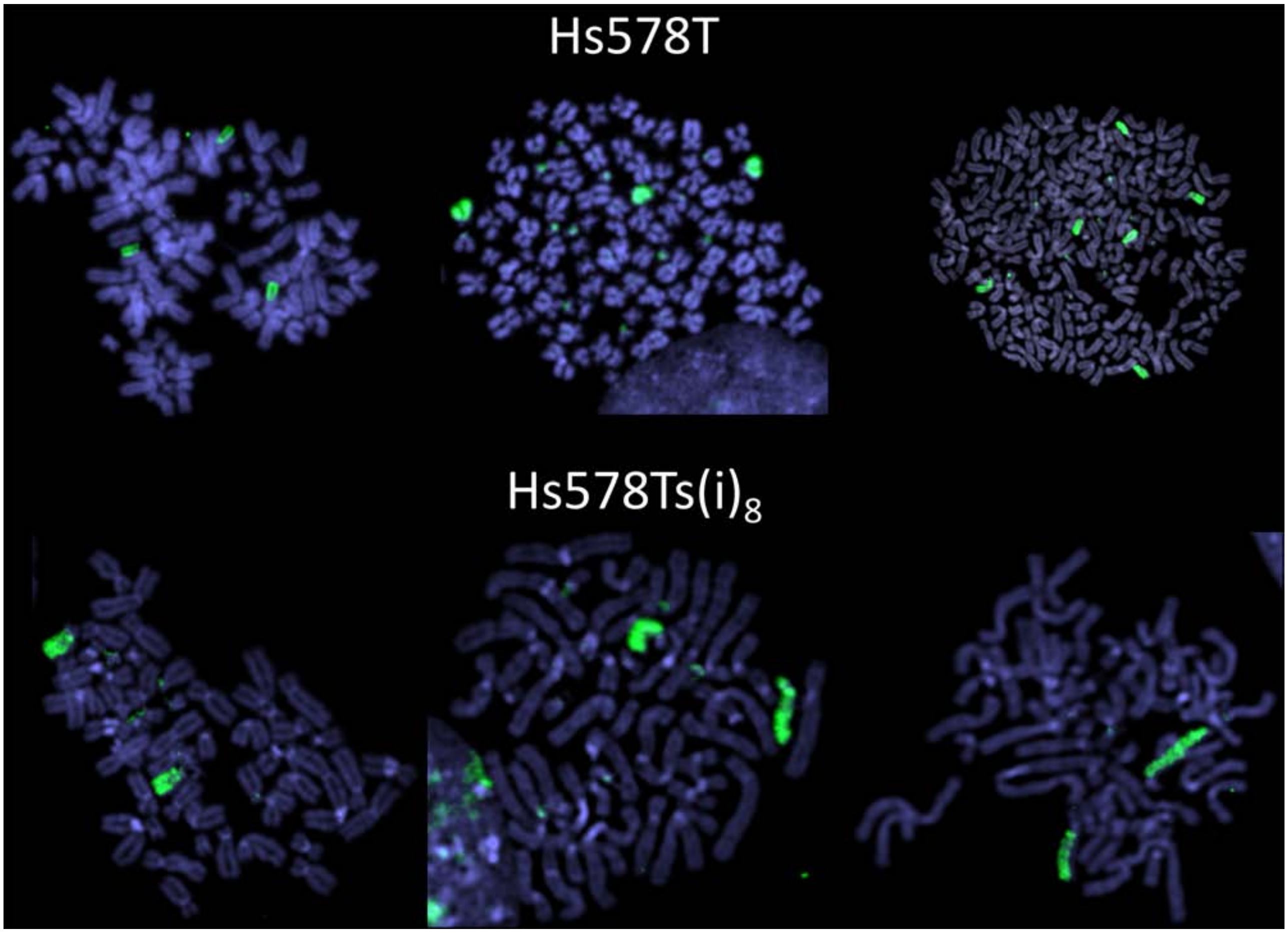

Supplementary Figure S2: Chromosome painting. Shows triploid/hexaploid (Hs578T) and quasi-diploid (Hs578Ts(i)$_8$) ploidies of the two sublines. Note loss of one copy of chromosome 14 in the Hs578Ts(i)$_8$ variant. Additional green signals represent cross hybridization onto ribosomal RNA genes present on short arms of chromosomes 13, 14 and 15.

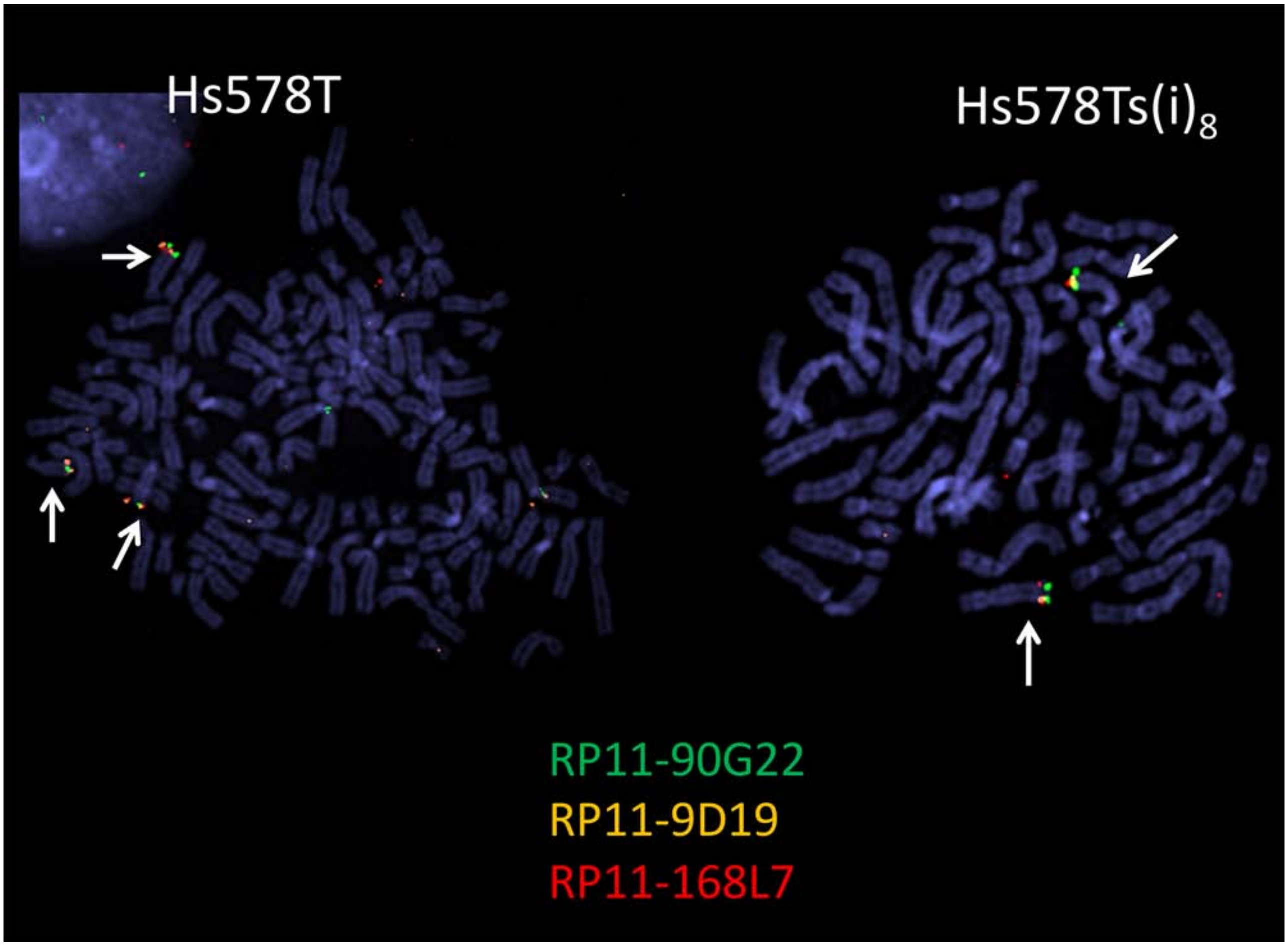

Supplementary Figure S3: FISH using BAC clones yielded intact triple red, green and yellow signals at all 14q32 loci. Hence, both cell line variants show un-rearranged ROI.

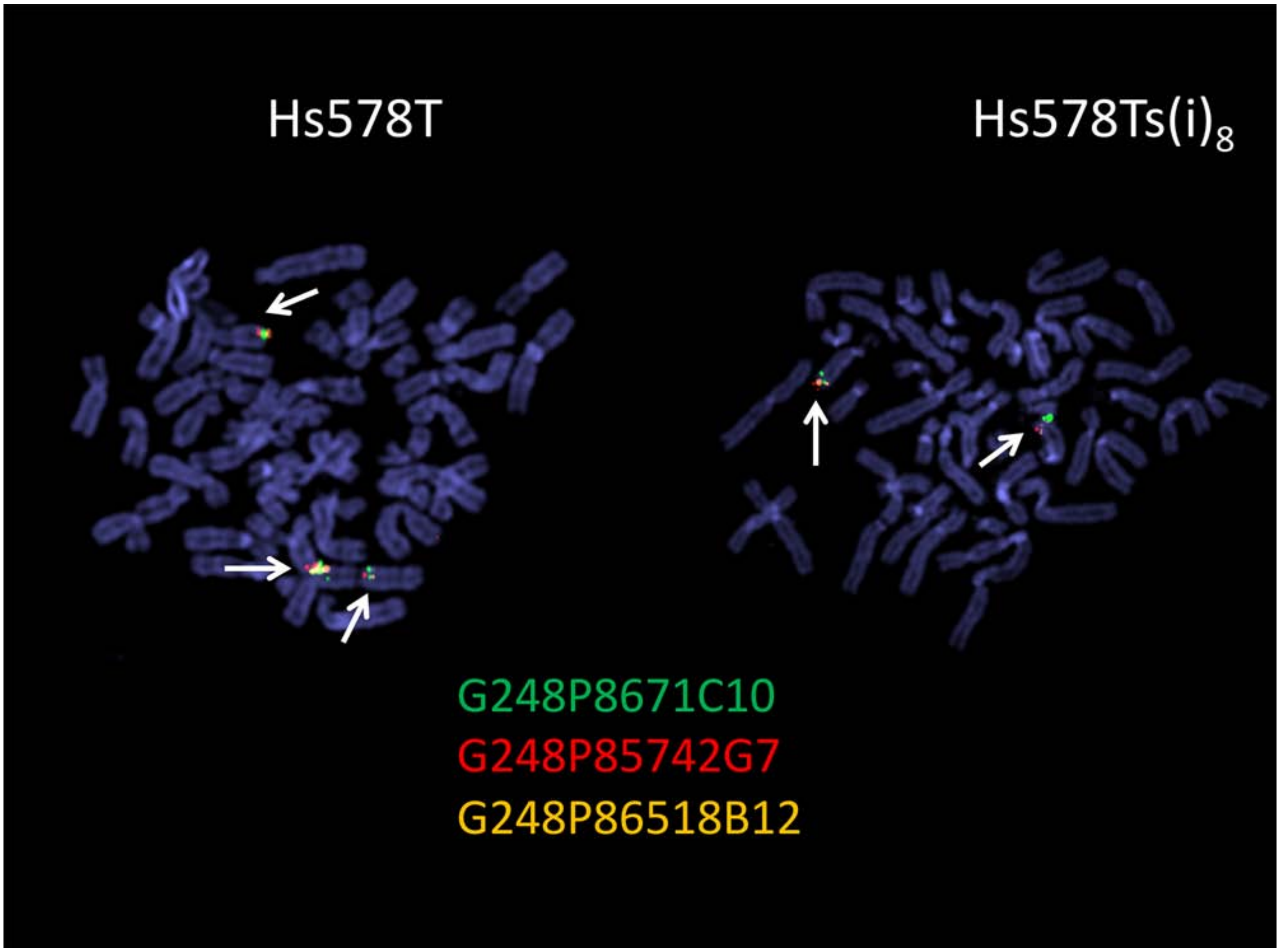

Supplementary Figure S4: FISH using fosmid clones also yielded intact signals. Note un-rearranged ROIs in both Hs 578T and Hs578Ts(i)$_8$.

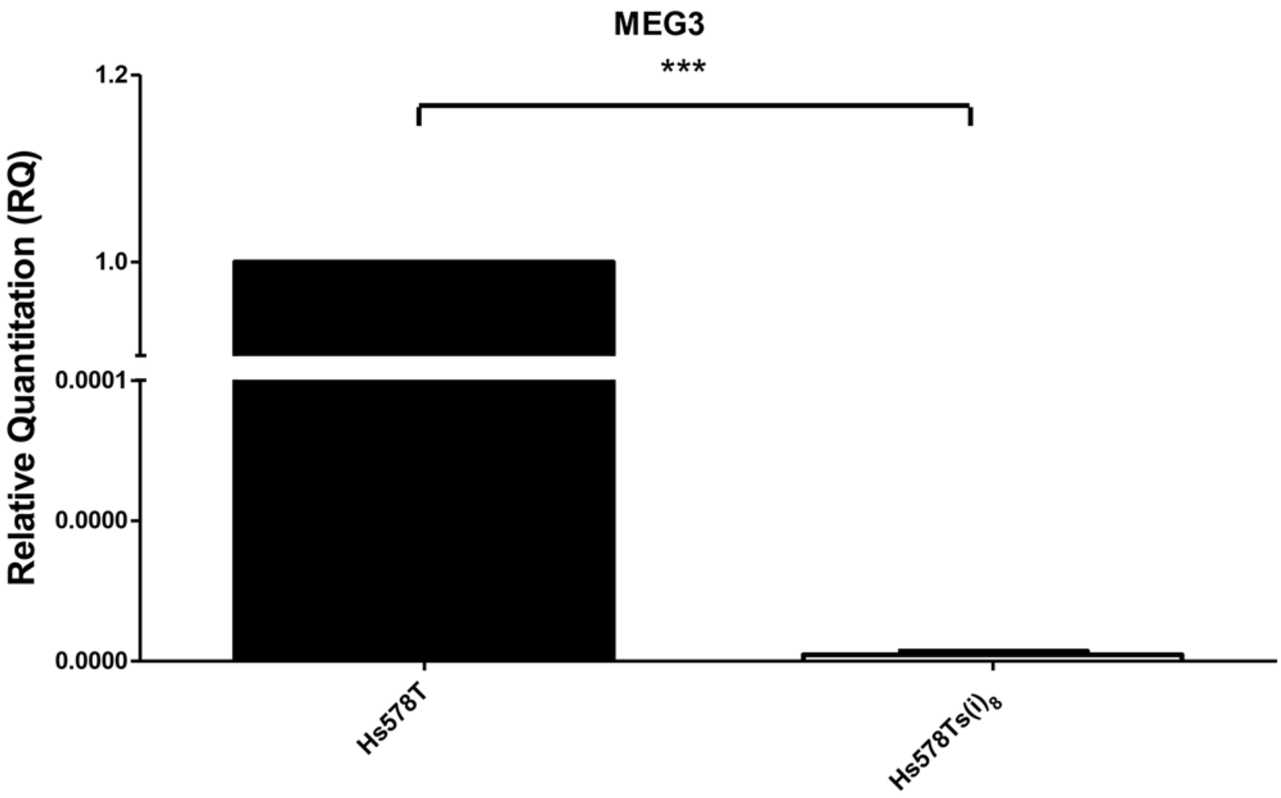

Supplementary Figure S5: Expression of MEG3 in Hs578T and Hs578Ts(i)$_8$ cells, evaluated using qPCR.

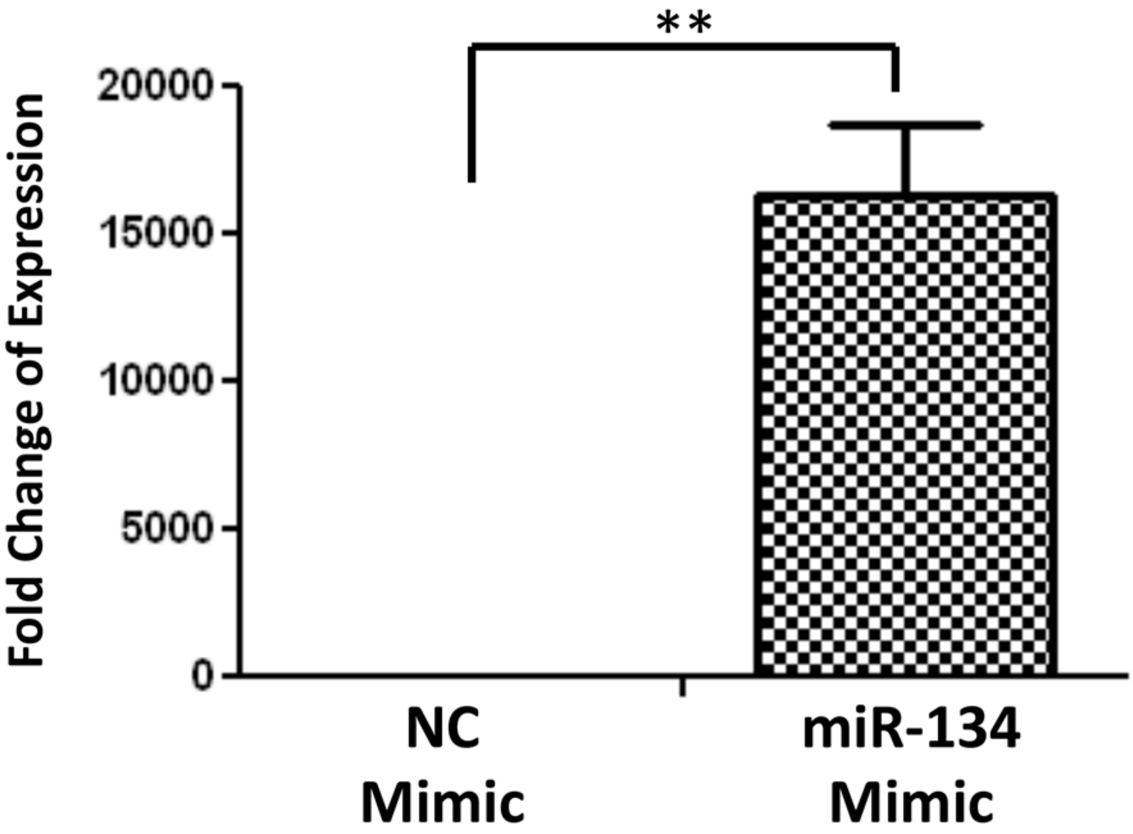

Supplementary Figure S6: Expression of miR-134 in Hs578Ts(i)$_8$ cells post-transfection with miR-134-mimic compared to the levels of miR-134 in NC-mimic transfected Hs578Ts(i)$_8$ cells, as determined by qPCR.

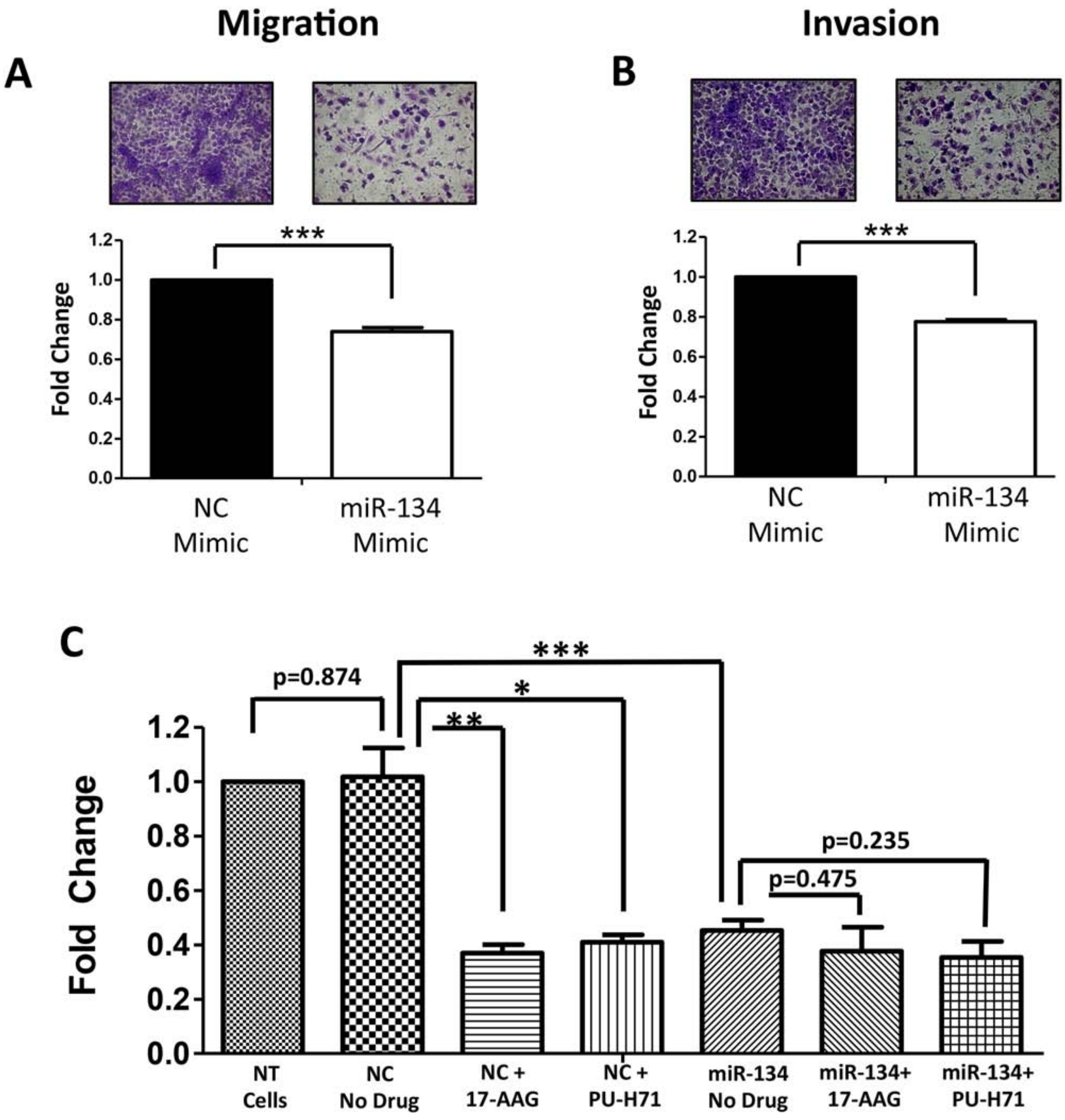

Supplementary Figure S7: Effect of miR-134 direct transfection on Hs578Ts(i)$_8$ migration, invasion and anti-Hsp90 drug sensitivity. Effect of miR-134 on Hs578Ts(i)$_8$ **A.** migration and **B.** invasion compared to the effects of NC-mimic transfection. **C.** 17-AAG or PU-H71 do not significantly increase the anti-proliferative effects of miR-134 on Hs578Ts(i)$_8$ cells, evaluated using acid phosphatase assays.

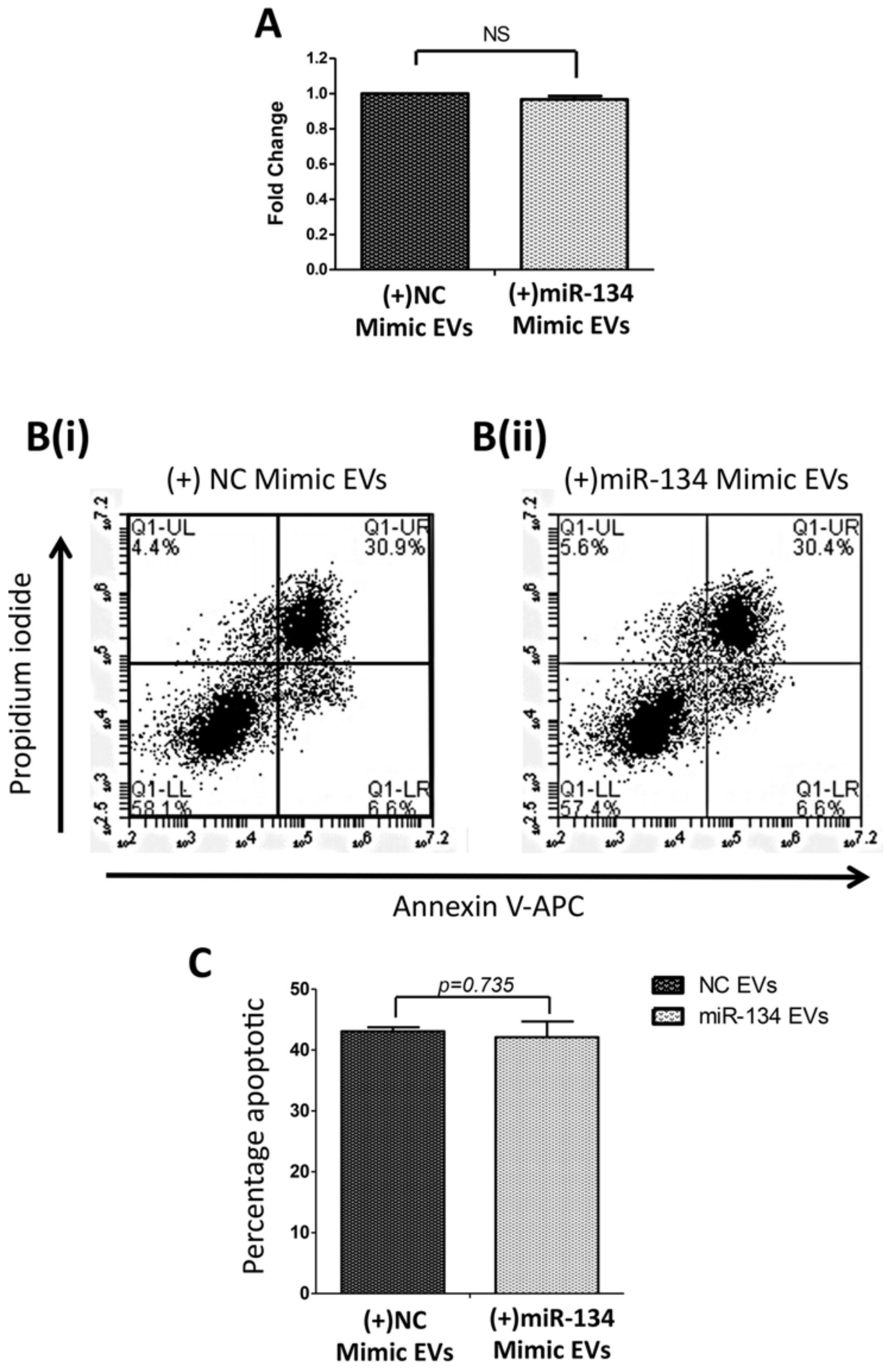

Supplementary Figure S8: Effect of miR-134-enriched EVs on Hs578Ts(i)$_8$ proliferation and cisplatin-induced apoptosis. A. miR-134-enriched EVs do not significantly alter Hs578Ts(i)$_8$ cell proliferation compared to the effects of EVs from NC-mimic transfected cells, assessed using acid phosphatase assays. **B.** Apoptosis analysis, by FACS, using Annexin-V APC and PI staining show that miR-134-enriched EVs do not significantly alter cisplatin-induced apoptosis compared to the effects of EVs from NC-mimic transfected cells. **B(i).** Representative scatter plots for NC-mimic derived EVs with cisplatin and **B(ii).** miR-134 enriched EVs with cisplatin. **C.** Graphical representation of total apoptosis observed.

Supplementary Table S1: miRNAs commonly down-regulated in both Hs578Ts(i)$_8$ cells and their EVs compared to Hs578T cells and their EVs, respectively.

miR-370	miR-204	miR-518f	miR-183
miR-379	miR-376a	miR-520f	miR-145
miR-382	miR-486–5p	miR-494	miR-143
miR-411	miR-654–3p	miR-140–3p	miR-372
miR-376c	miR-433	miR-182	miR-24
miR-495	miR-515–3p	miR-519a	miR-195
miR-134	miR-512–5p	miR-23a	miR-199b-5p
miR-127–3p	miR-487a	miR-515–5p	miR-135b
miR-889	miR-375	miR-138	miR-500
miR-655	miR-371–3p	miR-486–3p	miR-323–3p
miR-487b	miR-299–5p	miR-31	miR-221
miR-516b	miR-518a-3p	miR-34c-5p	miR-199a-5p
miR-519e	miR-526b	miR-148b	miR-28–5p
miR-493	miR-525–3p	miR-518d-5p	miR-27a
miR-337–5p	miR-518c	miR-532–5p	miR-185
miR-410	miR-518e	miR-517a	miR-130b
miR-485–3p	miR-512–3p	miR-140–5p	miR-522
miR-519c-3p	miR-520g	miR-135a	miR-28–3p
miR-654–5p	miR-519d	miR-523	miR-152
miR-431	miR-517c	miR-324–5p	miR-27b
miR-539	miR-362–5p	miR-29c	

miRNAs as listed based on fold change observed in Hs578Ts(i)$_8$ cells compared to Hs578T cells; Results represent three biological repeats.
